# Enhancing Lithium–Sulfur Battery Performance with MXene: Specialized Structures and Innovative Designs

**DOI:** 10.1002/advs.202404328

**Published:** 2024-07-25

**Authors:** Fei Li, Shijie Mei, Xing Ye, Haowei Yuan, Xiaoqin Li, Jie Tan, Xiaoli Zhao, Tongwei Wu, Xiehang Chen, Fang Wu, Yong Xiang, Hong Pan, Ming Huang, Zhiyu Xue

**Affiliations:** ^1^ School of Materials and Energy University of Electronic Science and Technology of China Chengdu 611731 China; ^2^ Frontier Center of Energy Distribution and Integration Tianfu Jiangxi Lab Chengdu 641419 China; ^3^ School of Materials Science and Engineering Xihua University Chengdu 610039 China; ^4^ Institute of Fundamental and Frontier Sciences University of Electronic Science and Technology of China Chengdu 611731 China

**Keywords:** catalysis, external functional groups, heterojunction, lithium‐sulfur batteries, MXene

## Abstract

Established in 1962, lithium–sulfur (Li–S) batteries boast a longer history than commonly utilized lithium–ion batteries counterparts such as LiCoO_2_ (LCO) and LiFePO_4_ (LFP) series, yet they have been slow to achieve commercialization. This delay, significantly impacting loading capacity and cycle life, stems from the long‐criticized low conductivity of the cathode and its byproducts, alongside challenges related to the shuttle effect, and volume expansion. Strategies to improve the electrochemical performance of Li–S batteries involve improving the conductivity of the sulfur cathode, employing an adamantane framework as the sulfur host, and incorporating catalysts to promote the transformation of lithium polysulfides (LiPSs). 2D MXene and its derived materials can achieve almost all of the above functions due to their numerous active sites, external groups, and ease of synthesis and modification. This review comprehensively summarizes the functionalization advantages of MXene‐based materials in Li–S batteries, including high‐speed ionic conduction, structural diversity, shuttle effect inhibition, dendrite suppression, and catalytic activity from fundamental principles to practical applications. The classification of usage methods is also discussed. Finally, leveraging the research progress of MXene, the potential and prospects for its novel application in the Li–S field are proposed.

## Graphical abstract

This review summarizes the advantages and recent advancements of MXene‐based materials in Li–S batteries, including high‐speed ionic conduction, structural diversity from its 2D structure, functional groups that suppress the shuttle effect, catalytic activity, and dendrite inhibition. It spans from foundational principles to practical applications and highlights the potential and future prospects of MXene‐based materials in the Li–S field.

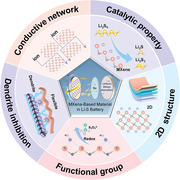

## 1 Introduction

The landscape of the power industry, traditionally based on non‐renewable fossil energy sources, is undergoing rapid transformation, heralding the era of electrified vehicles. There is a heightened demand in the market for energy storage devices with high energy density and prolonged cycle life.^[^ ^1^ ^]^ Li–S batteries have garnered significant attention owing to the impressive theoretical capacity of sulfur (1675 mAh/g) and high energy density (2600 Wh kg^−1^).^[^ ^2^ ^]^ Moreover, the favorable attributes of sulfur, characterized by its low cost, ready availability, and environmental friendliness, have sparked widespread interest.

Nevertheless, the pragmatic implementation of Li–S batteries is confronted with a spectrum of challenges: (i) Shuttle effect of soluble intermediate polysulfides: the presence of soluble intermediate products, notably long‐chain Li_2_S_n_ (4 ≤ n ≤ 8), instigates the shuttle effect, leading to pronounced capacity deterioration and a curtailed cycle life.^[^ ^3^ ^]^ (ii) Insulativity of Sulfur and Li_2_S: the intrinsic insulating properties of sulfur and Li_2_S contribute to inadequate sulfur utilization and suboptimal rate capabilities. (iii) Substantial Volume Change of S/Li_2_S (80%): during the phase transition from sulfur to Li_2_S, a substantial volumetric expansion of ≈ 80% is observed, which poses a significant risk to the structural integrity of the electrode. (iv) Formation of lithium dendrites: the occurrence of lithium dendrites on the anode during the charging and discharging (C&D) processes introduces the risk of short‐circuiting, further complicating the viability of Li–S batteries in practical applications.^[^ ^4^ ^]^ Considerable endeavors have been devoted in recent decades to surmount the enumerated challenges. These endeavors encompass diverse methodologies, such as the physical confinement of sulfur within porous carbons,^[^ ^3^, ^5^ ^]^ chemical tethering of LiPSs through polar metal compounds,^[^ ^6^ ^]^ the introduction of a conductive interlayer at the cathode‐separator interface,^[^ ^7^ ^]^ and the engineering of advanced Li anodes to suppress dendritic growth.^[^ ^8^ ^]^ Despite the partial amelioration achieved through these strategies, the attainment of broad commercial viability for Li–S batteries remains elusive.

MXene was discovered in 2011 by Prof. Yury Gogotsi and Prof. Michel Barsoum.^[^ ^9^ ^]^ The general formula is M_n+1_X_n_T_x_ (n = 1, 2, 3),^[^ ^10^ ^]^ in which M refers to the early transition metal (TM) elements (e.g., Zr, Ti, Sc, V, Nb, Mo, Hf, etc.), X denotes carbon (C), nitrogen (N), or a certain proportion of C and N combinations, and T generally represents the functional group modifiers (e.g., ‐OH, ‐O, and ‐F) located on the surface of the nanosheets.^[^ ^11^ ^]^ MXene materials have the main characteristics of 2D structure, metallic conductivity, highly active surface, and water dispersion.^[^ ^12^ ^]^ Therefore, they have been widely studied in many fields such as energy storage,^[^ ^13^ ^]^ electrocatalysts,^[^ ^14^ ^]^ electromagnetic interference shielding,^[^ ^15^ ^]^ and biomedicine,^[^ ^16^ ^]^ especially in various energy storage devices such as lithium–ion batteries,^[^ ^17^ ^]^ Li–S batteries,^[^ ^18^ ^]^ zinc‐ion batteries,^[^ ^19^ ^]^ sodium‐ion batteries,^[^ ^20^ ^]^ supercapacitors,^[^ ^13^, ^21^ ^]^ etc.

Conspicuously, MXene shows up‐and‐coming applications in achieving practical Li–S batteries. Its metallic conductivity offers faster electron transfer and increases the full utilization of sulfur, thus ensuring the high capacity of Li–S batteries.^[^ ^22^ ^]^ MXene, with its 2D lamellar structure, is an ideal material for loading sulfur as the cathode.^[^ ^23^ ^]^ The termination layer on the surface can strongly adsorb LIPSs and form robust metal‐S bonds, preventing the shuttle effect.^[^ ^24^ ^]^ In addition, MXene with abundant termination layers are highly efficient in catalyzing the conversion between LIPSs and Li_2_S, thus facilitating the redox kinetics during cycling.^[^ ^25^ ^]^ MXene is also related to uniform nucleation and growth of lithium,^[^ ^26^ ^]^ thereby inhibiting dendrite. Furthermore, the above features can be swiftly realized and modified through grafting and compositing.^[^ ^27^ ^]^

Herein, this comprehensive review offers insight into the functionalization advantages that MXene‐based materials bring to Li–S batteries. We delve into the benefits of MXene, encompassing high‐speed ionic conduction, structural diversity, mitigation of the shuttle effect, dendrite suppression and catalytic activity, spanning from fundamental principles to practical applications. Additionally, we investigate the classification of practical methodologies and offer a comprehensive understanding of MXene. Finally, drawing upon the state‐of‐art advancements in MXene research, the potential applications of its innovative discoveries are proposed. In conclusion, this synthesis of existing knowledge contributes to a deeper comprehension of how MXene could significantly propel the advancement of Li–S battery technology.

## 2 Functionalization of MXene‐Based Materials in Lithium–Sulfur Batteries

Following the research boom of graphene, its MXene brothers have also received widespread attention. While possessing conductivity, 2D structure, and interface properties comparable to Graphene, it has unique polar catalysis and bonding ability caused by TM elements and polar groups.^[^ ^28^ ^]^ Moreover, compared to conventional catalysts, MXene has better physical structural advantages,^[^ ^29^ ^]^ making it an ideal strategy for solving problems related to Li–S batteries.

### 2.1 Conductivity Applications

Li–S batteries are highly promising energy storage devices today. However, the poor conductivity of the active substance sulfur and the discharge products have slowed down the practical application.^[^ ^3^, ^30^ ^]^ The insulating properties lead to a decrease of active materials utilization and performance deterioration. MXene can neutralize this drawback due to their intrinsic electrical conductivity.^[^ ^9^, ^31^ ^]^ As reported, pure Ti_3_C_2_T_x_ films display electrical conductivities as high as 10^5^ S m^−1^.^[^ ^32^ ^]^ Elements like Ti, V, W, Ta, Hf on the M site are weighted in structural stability and conductivity. MXene's high conductivity promotes rapid electron transfer and sulfur conversion, which facilitates the full utilization of sulfur, and increases the final discharge capacity value.^[^ ^33^ ^]^ Overview of numerous studies on conductive Li–S battery, which can be classified into two types: direct application of MXene and combing MXene with other materials.

#### 2.1.1 Direct Application of MXene

**Figure 1:**
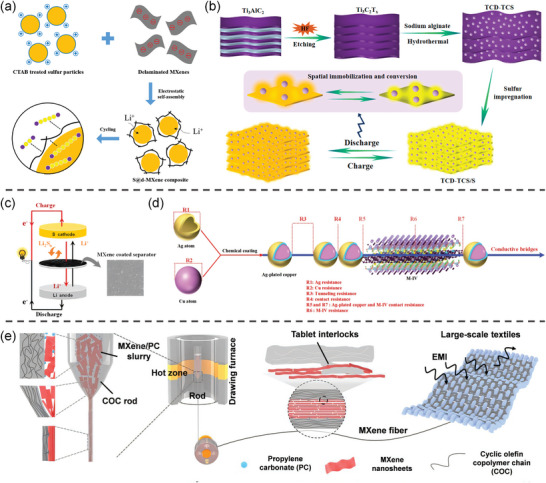
a) Schematic of the fabrication of S@d‐MXene‐wrapped sulfur composites. Reproduced with permission.^[^ ^35a^ ^]^ Copyright 2022, Elsevier. b) The schematic diagram for the preparation of TCD‐TCS and TCD‐TCS/S. Reproduced with permission.^[^ ^36^ ^]^ Copyright 2019, American Chemical Society. c) Schematic configuration of the Li–S cells using PP and MPP separators. Reproduced with permission.^[^ ^37b^ ^]^ Copyright 2016, American Chemical Society. d) The compositing process and resistance components of conductive media. Reproduced with permission.^[^ ^39^ ^]^ Copyright 2023, Wiley‐VCH. e) Schematic diagram of fabrication of MXene fiber with tablet interlocks. Reproduced with permission.^[^ ^38^ ^]^ Copyright 2023, Wiley‐VCH.

MXene with high electrical conductivity can be used as a cathode material for Li–S batteries to load the active substance sulfur,^[^ ^22^, ^34^ ^]^ thereby solving the sulfur insulation issue. When employed as sulfur loader, MXene encapsulate sulfur as a thin sheet or uniformly load sulfur as a layered carrier. The poor electrochemical properties of sulfur can be greatly improved by wrapping sulfur particles in 2D MXene flakes.^[^ ^35^ ^]^ Lee et al. prepared the delaminated MXene‐wrapped sulfur composite (S@d‐MXene) by a simple electrostatic self‐assembly method of combining positively charged elemental sulfur and negatively charged D‐MXene (**Figure** **1**a), which endowed the sulfur with high electrical conductivity.^[^ ^35a^ ^]^ Comparing the Nyquist plots, the cell with S@d‐MXene composite cathode exhibits a much lower charge‐transfer resistance (Rct) (65 Ω) than regular cathode (290.1 Ω). D‐MXene can enhance the conductivity of sulfur by providing a fast pathway for ions and electrons in the electrode. The synthesis method of MXene affects how uniformly the sulfur is loaded, which in turn alters conductivity. Xiao et al. hydrothermally treated the prepared Ti_3_C_2_T_x_ with sodium alginate at 100 °C for 2 h to obtain Ti_3_C_2_T_x_ nanodots‐interspersed Ti_3_C_2_T_x_ nanosheet (TCD‐TCS) (Figure 1b), which has a conductivity as high as 1320 ± 50 S cm^−1^.^[^ ^36^ ^]^ In this battery, the active sulfur is in close contact with TCD‐TCS, the diffusion distance of electrons and lithium–ion (Li^+^) is short, and the electrode material can be better wetted by the electrolyte. These greatly weaken the insulating property of sulfur and favor the electrochemical reaction.

The ultrafast conductive channels and electron transfer pathways are crucial for conductive materials and power batteries. For example, MXene materials can be used as a functional layer of a diaphragm.^[^ ^37^ ^]^ Song et al. reported the enhancement of the electrochemical performance of Li–S batteries by coating Ti_3_C_2_T_x_ MXene nanosheets onto commercial “Celgard” membrane (Figure 1c).^[^ ^37b^ ^]^ The electrochemical impedance spectroscopy (EIS) shows that the Rct of the celgard to modified separator is reduced from 101.2 Ω to 45.06 Ω. MXene composited with Ag‐plated copper powder was selected as the conductive highly‐conductive media which has greatly reduced the contact resistance and tunneling resistance, and reached a resistivity of 9.668 × 10^−7^ Ω m (1.03 × 10^6^ S m^−1^) (Figure 1d).

In addition, some processing methods such as hot stretching, freeze‐drying, and etching can further modify the conductive properties of MXene. Wei et al. prepared outstanding conductivity and ultra‐long MXene fibers, using fluidics‐assisted thermal drawing technology by in situ generating a highly oriented copolymer protective layer (Figure 1e).^[^ ^38^ ^]^ Such method can avoid loose structure of multilayer and obtain MXene that is more practical in batteries. Furthermore, better Li^+^ transfer can be achieved by adjusting the pore size to give MXene a perforated structure. Xiong et al. prepared porous MXene materials by oxidative etching and realized the adjustment of pore size by adjusting the concentration of catalyst Cu^2+^.^[^ ^37c^ ^]^

**Figure 2:**
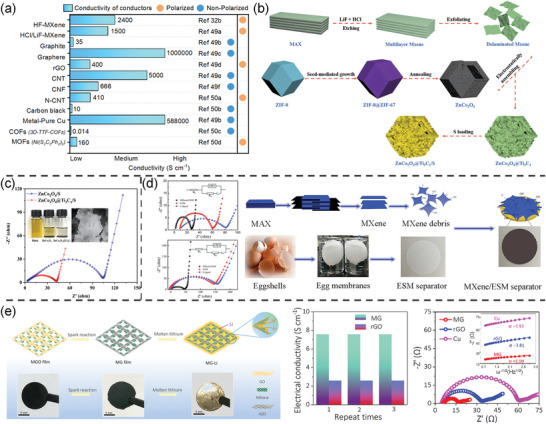
a) Comparison of conductivity and polarity of commonly used conductive materials for Li–S batteries.^[^ ^32^, ^49^, ^50^ ^]^ b) Illustration of the formation process of the ZnCo_2_O_4_@Ti_3_C_2_/S. c) Nyquist plots of ZnCo_2_O_4_@Ti_3_C_2_/S and ZnCo_2_O_4_/S after 100 cycles at 0.2 C. Reproduced with permission.^[^ ^44c^ ^]^ Copyright 2022, Elsevier. d) Nyquist plots before and after 100 cycles with the Celgard, ESM, and MXene/ESM; and scheme of preparation process of the MXene/ESM. Reproduced with permission.^[^ ^46^ ^]^ Copyright 2018, Elsevier. e) Schematic illustration of the fabrication process of the 3D MG‐Li anode and corresponding photographs of the MGO film, MG film, and MG‐Li anode; electrical conductivity of an MG and a pure rGO electrode; and Nyquist plots of MG, rGO, and Cu foil electrodes. Reproduced with permission.^[^ ^48b^ ^]^ Copyright 2019, American Chemical Society.

The results demonstrate that the MXene nanosheet coating can significantly reduce the resistance of the cell due to its high electronic conductivity, thereby promoting faster redox kinetics and enhancing the electrochemical performance of the battery. Compared to ordinary conductors, MXene exhibits excellent conductivity and polarity characteristics to modify Li–S batteries (**Figure** **2**a). The use of MXene as a modification material can improve Li^+^ transport efficiency, accelerate polysulfide redox reactions, and increase sulfur utilization.

#### 2.1.2 MXene‐Based Composites

The functionality of a single material is always limited. As the use of MXene materials for Li–S battery became better understood, researchers began combining them with other superior materials.^[^ ^40^ ^]^ For example, MXene materials have a tendency to self‐stack, and compounding VS_4_ on their surfaces effectively prevents this tendency and improves ion transport efficiency.^[^ ^41^ ^]^ Furthermore, both MXene and carbon nanotube (CNT) are good conductive materials. Wrapping CNT with MXene flakes not only combines the advantages of high conductivity of both, but also solves the problem of self‐stacking of MXene.^[^ ^40c^ ^]^

MXene materials are often compounded with other materials as a sulfur cathode,^[^ ^23^, ^42^ ^]^ such as metal oxides and sulfides.^[^ ^43^ ^]^ The chemisorption sites of metal oxides and sulfides can effectively chemisorb LIPSs, promote their rapid conversion.^[^ ^44^ ^]^ However, their poor electronic conductivity limits their application in sulfur cathode materials.^[^ ^45^ ^]^ Wei et al. obtained ZnCo_2_O_4_ and Ti_3_C_2_ composites by simple electrostatic self‐assembly method and use them as sulfur‐host. The EIS show that the ZnCo_2_O_4_@Ti_3_C_2_/S composite has a lower Rct and higher conductivity compared to the ZnCo_2_O_4_/S cathode (Figure 2b).^[^ ^44c^ ^]^ Yin et al. obtained high‐performance separators by coating eggshell membranes with MXene debris.^[^ ^46^ ^]^ The EIS before and after cycling revealed that the eggshells‐membranes (ESM)/MXene separator was able to provide fast charge transfer than Celgard and ESM separators (Figure 2c), which was attributed to the ultrafast conduction network within the MXene debris. In addition, using MXene as a conductive substrate and growing polar semiconductors on it is also a promising strategy for separator modification.^[^ ^41^, ^47^ ^]^

The application of MXene to Li–S battery anodes has also been reported.^[^ ^48^ ^]^ For example, Shi et al. reported a highly conductive, lithophilic, lightweight MXene/graphene (MG) framework for Li–S battery anodes.^[^ ^48b^ ^]^ As shown in Figure 2d, the conductivity of the MG film is 7.6 S cm^−1^, much higher than reduced graphene oxide (rGO) film. The interfacial Rct of the MG is low. The MXene‐modified surface exhibited Li coverage without dendritic growth, while the bare rGO region showed irregular Li dendrites. In conclusion, MXene material can fully utilize its advantage of good electrical conductivity to enable uniform deposition of Li and reduce the formation of Li dendrites.

To summarize, MXene is extensively used in Li–S batteries because of its superior conductive network and its Li^+^ affinity interface with polar compounds. It integrates well with other materials in Li–S batteries and serves as a versatile material for sulfur loading, separator modification, and as an anode material. Combining MXene with other materials is a strategic choice to capitalize on the strengths of different components.

### 2.2 Diverse Structural Advantages of MXene

MXene has a layered and porous structure, large surface area, metal conductivity, and an abundance of surface group. These properties have triggered significant research activities in a variety of fields, especially in the field of Li–S batteries.^[^ ^51^ ^]^

#### 2.2.1 Structural Advantages – High Sulfur Loading Capacity

The 2D structure provides ideal sites for sulfur loading.^[^ ^52^ ^]^ Even if the nanosheet structure tends to stack together, the optimization of intercalation and synthesis can easily solve it. Generally, when electron‐rich groups (‐Cl, ‐F, ‐OH) are grafted on the surface of MXene, the repulsive interaction of these groups is larger than the van der Waals (vdW) forces and hydrogen bonding between the layers, which increases the distance between the layers. In other words, the surface of MXene is filled with variables and operable upgrade space. 2D MXene can be easily constructed into specialized pattern, such as porous nanoribbons, frameworks, and composite aerogel,^[^ ^51^, ^53^ ^]^ which provide ample active sites for sulfur and great scope for high sulfur loading.

The porous structure of MXene allows it to be inserted to accommodate a large number of sulfur atoms. For example, the TCD‐TCS cathode shows a high BET surface area of 138 m^2^ g^−1^, and its pore size is mainly composed of 3 nm mesopores. The area specific loading of sulfur was as large as 9.2 mg cm^−2^.^[^ ^36^ ^]^ Zhang et al. introduced a lamellar MXene/CNT aerogel by unidirectional freeze‐drying to enhance the cycling stability for high sulfur loading Li–S batteries.^[^ ^53b^ ^]^ The MXene/CNT sandwiches based Li–S battery provided a high capacity of 712 mAh g^−1^ at a sulfur content of 7 mg cm^−2^. Generally, after alkaline immersion of MXene and oscillatory treatment, 2D Ti_3_C_2_ MXene can be delaminated and split into MXene nanoribbons,^[^ ^48a^ ^]^ which expose most of the pores inside. In addition, co‐melting is also a commonly used method for preparing S/MXene cathodes. Dong et al. prepared S/MXene cathode with a sulfur content of 57.6 wt% using direct melt‐diffusion process.^[^ ^51d^ ^]^ The performance of the cathode is higher (70 wt%) when it is subsequently treated with dimethyl sulfoxide (DMSO) immersing, which laminate accordion shaped MXene into thinner nanosheets. To sum up, nanoribbon possessing interconnected porous frameworks, which can work as S/polysulfides host and coating protective layer.^[^ ^28^, ^54^ ^]^

Surface modification is an essential strategy to increase sulfur loading by suppressing the stacking of MXene nanosheets. Density functional theory (DFT) calculations were used to predict the bond force of different groups with LIPSs, thereby guiding the selection of ideal sulfur host.^[^ ^51d^ ^]^ Liu et al. applied DFT to investigate the LIPSs anchoring properties of Ti_3_C_2_ MXene with different end groups (‐OH, ‐F, ‐S).^[^ ^28^ ^]^ The functional groups grafted on the surface of MXene will undergo complex reactions with LIPSs, thereby affecting the bonding situation of the initial interface (e.g., Ti‐S bonding).

Heteroatom doping is another method to enhance its sulfur loading of MXene, which can improve its original physicochemical properties.^[^ ^48a^ ^]^ Direct etching with ZnCl_2_ in the molten state allows injection of Zn atoms into Ti atom sites on the surface, attracting a portion of the polysulfide due to the high electronegativity of the Zn atoms.^[^ ^55^ ^]^ After loading sulfur, Zn‐implanted MXene electrode can achieve 89 wt% sulfur content. Wang et al. used the Mo_2_SnC system with Sn removed to obtain the MXene structure.^[^ ^56^ ^]^ During the discharge process, the powerful bonding of highly active Mo atoms and strong electronegative sulfur ions can load more sulfur (87.1 wt% sulfur content). Furthermore, non‐metallic atom doping was used to adjust the affinity of LIPSs and Li^+^ diffusion. For example, 3D wrinkled N‐doped Ti_3_C_2_T_x_ MXene frameworks can be prepared by heat‐treating with a mixture of Ti_3_C_2_T_x_ flakes and melamine, and its sulfur loading can reach 78.3 wt%,^[^ ^57^ ^]^ suggesting that the N doped MXene has outstanding ability to load sulfur.

The sulfur loading of Li–S pouch batteries is usually 5 mg cm^−2^ or more, with MXene‐based sulfur hosts are ranking in the upper‐middle range of high loadings.^[^ ^58^ ^]^ For example, the N/CF@V_2_CT_x_ host achieves over 6 mg cm^−2^, compared 1.5 mg cm^−2^ in coin button cells.^[^ ^59^ ^]^ The electrolyte volume to the sulfur mass of Li–S cells is less than 5 µL mg^−1^, whereas it is usually 16 µL mg^−1^ in coin cells. Li–S cells face significant degradation issues, with over 30% of the capacity loss in Ah‐grade pack batteries resulting from severe metal surface corrosion and successive volume changes.^[^ ^60^ ^]^ MXene, which introduces more active sites and serves as an interfacial protective layer for the electrodes, has proven to be highly effective in Li–S utility studies.

#### 2.2.2 Interface Performance – Heterojunctions

**Figure 3:**
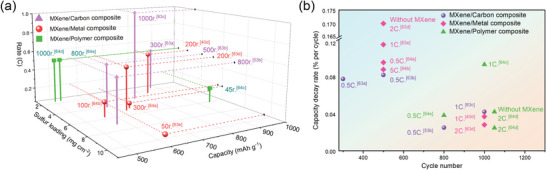
a) 3D comparison chart of discharge capacity, rate, and sulfur loading of commonly used heterojunctions. b) Capacity decay rate of MXene‐based batteries with normal sulfur loading (<2 mg cm^−3^). Reproduced with permission.^[^ ^40^, ^53^, ^63^, ^64^ ^]^

Since 2011, many researchers paid attention to MXenes heterojunction with considerable internal space and binding sites.^[^ ^61^ ^]^ In this section, we review three common MXene heterojunctions, namely MXene/Carbon,^[^ ^62^ ^]^ MXene/metal, and MXene/polymer.^[^ ^61b^ ^]^ **Figure** **3**a shows the MXene‐based heterojunction for sulfur hosts. The MXene/carbon composite shows great promise. For example, the point labeled “1000r.^[^ ^60c^ ^]^” indicates a battery capacity of 755 mAh/g after 1000 cycles at 1C. However, the low density and high volume occupancy of carbon make achieving high sulfur loading difficult, limiting it to only 3.2 mg cm^−2^. By adding MXene to form a heterojunction, the rate of capacity decay in the battery is significantly reduced (Figure 3b).

The MXene/C heterojunction is the most common form. We categorize common carbon‐based heterostructures into 1D, 2D, and lower scale materials according to their dimensionality. Generally speaking, 1D heterojunction often includes CNT^[^ ^65^ ^]^ and Carbon Nanofiber (CNF),^[^ ^66^ ^]^ 2D heterojunction usually includes grapheme, and porous carbon (including mesoporous carbon and covalent organic framework (COF)),^[^ ^67^ ^]^ 0D heterojunction mainly includes hollow carbon nanospheres.

**Figure 4:**
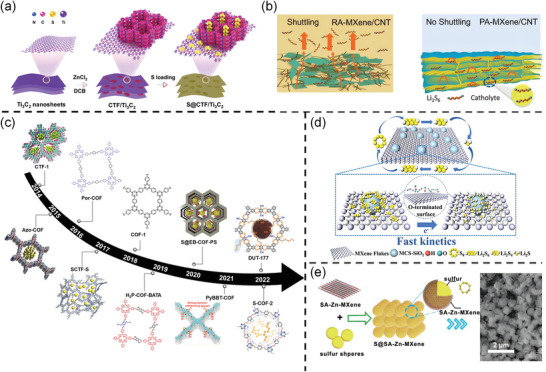
a) Schematic illustration of the synthetic process of the 2D CTF/TNS heterojunction and the S@CTF/TNS composites. Reproduced with permission.^[^ ^42a^ ^]^ Copyright 2020, Wiley‐VCH. b) The schematic mechanism of Li–S batteries with PA‐MXene/CNT and RA‐MXene/CNT. Reproduced with permission.^[^ ^53b^ ^]^ Copyright 2021, Wiley‐VCH. c) An evolution timeline of the COF‐based materials for use in Li–S batteries. Reproduced with permission.^[^ ^79^ ^]^ Copyright 2023, Wiley‐VCH. d) Schematic illustration of the conversion of LIPSs in S@MCS‐SiO_2_/MXene cathode. Reproduced with permission.^[^ ^63c^ ^]^ Copyright 2022, Elsevier. e) The illustration of the fabrication of SA‐Zn‐MXene coated sulfur. Reproduced with permission.^[^ ^55a^ ^]^ Copyright 2020, Wiley‐VCH.

i) The introduction of 1D materials like CNT, CNF into MXene not only acts as a spacer insertion agent to prevent the stacking of the nanosheets but also enables fast crosslinking electron transport.^[^ ^68^ ^]^ Accessing CNT heterojunction in MXene has played such a role (**Figure** **4**a). Nazar's group incorporated CNT as sulfur hosts into the MXene phase by interweaving CNT into the MXene layer (Figure 4b).^[^ ^36^ ^]^ In contrast, CNF usually plays a skeleton‐like role when accessing the heterojunction, serving as a carrier for introducing other materials. Li et al. synthesized Co‐based MOF (metal organic framework) in situ on CNF to form encapsulated composite structures.^[^ ^69^ ^]^ Lei et al. used an efficient one‐step hydrothermal reaction to obtain polar WS_2_ nanosheets deposited on CNF.^[^ ^70^ ^]^ These 3D fiber networks establish an interconnected conductive framework, facilitating rapid electron transfer during C&D so as to obtain excellent rate and cycling performance.
ii) GO and mesoporous carbon are the two widely used 2D carbon materials, which were usually combined with MXene to fabricate cathodes in Li–S battery.^[^ ^71^ ^]^ Du et al. employ the DFT to investigated the electrochemical properties of MG heterojunction.^[^ ^72^ ^]^ The calculation results show that the presence of graphene enhances the electrical conductivity, lithium adsorption and mechanical strength. Yang et al. prepared a porous aerogel structure with 3D conductive network by introducing GO into MXene to realize the rapid transfer of Li^+^ and electrons.^[^ ^66^ ^]^ Besides, highly ordered mesoporous carbons not only provide more space for sulfur loading and volume expansion, but also accelerate the encapsulation of LIPSs, allowing them to be employed for a wide range of energy conversion and storage.^[^ ^5b^ ^]^

COF, another 2D heterojunction, has generated a great deal of researches because of its ordered structural periodicity, inherent porosity, and abundance of functional ligands (Figure 4c). However, the low electrical conductivity of COF cannot meet the requirements for practical applications. Combining COF with conductive MXene is an effective method to improve the electrical conductivity. Yang's group reports a 2D–2D heterojunction consisting of in‐situ‐grown layered porous COF on Ti_3_C_2_‐MXene nanosheets.^[^ ^67b^ ^]^ This heterojunction provides a large surface area (318 m^2^ g^−1^) and abundant pore structure to load sulfur and withstand volume changes. In another report, guanidine ionic covalent organic nanosheets (iCON) were uniformly distributed on the surface of Ti_3_C_2_ nanosheets,^[^ ^73^ ^]^ and the palladium‐like Ti_3_C_2_@iCON heterojunction was prepared by the schiff base condensation reaction between the guanidine and the mesitylene. The positively charged guanidine unit traps the negatively charged LIPSs by electrostatic adsorption, thus inhibiting the migration of LIPSs to the anode.

iii) Lower scale carbon materials such as nanospheres and micro‐dots are flourishing. Specifically, Liang et al. prepared mesoporous carbon nanospheres (MCS‐SiO_2_), which are formed through self‐assembly and high‐temperature pyrolysis strategies, are securely embedded in the interlayer space of MXene nanosheets (Figure 4d).^[^ ^63c^ ^]^ MCS‐SiO_2_ trapped the polysulfides by physical confinement while mitigating cathodic volume expansion and collapse. The 0D functional points or balls embedded in the excellent layered host MXene can accelerate the diffusion of Li^+^ and guarantee the uniform deposition of Li_2_S.

MXene/metal is another type of heterojunction. In general, when preparing highly loaded sulfur cathodes, the carbon‐based composites tend to have thicker structures, while the metal‐based matrices tend to have higher grafting densities, which can load more sulfur.^[^ ^61e^ ^]^ Figure 3 also verified higher volumetric energy density of Li–S batteries with MXene/metal heterojunctions. They mainly include two categories, namely metal organic framework (MOF) and single atoms (SAs).

MOFs were ideal composite materials due to their large specific surface area, high porosity, appropriate pore size, open reaction centers, and heteroatom doping. Layered Zr‐MOF/Ti_3_C_2_T_x_ nanosheets were prepared by in situ solvothermal and electrostatic self‐assembly methods.^[^ ^74^ ^]^ The polyhedral‐shaped Zr‐MOF was located in the middle layer of MXene nanosheets, fully exposing the surface sites of MXene for electrochemical reactions. Its unique heterojunction has a large specific surface area of 911.5 m^2^ g^−1^ and a pore volume of 0.36 cm^3^ g^−1^.

MXene‐SAs heterojunction, with highly exposed active sites and maximum rate of atom utilization, can be used as the center for anchoring and electrocatalyzing LIPSs. TM elements with polarity and reactivity are ideal choices for single atom or hybrid single atom heterojunctions such as CoTe,^[^ ^75^ ^]^ FeSe_2_,^[^ ^61c^ ^]^ and V_2_C.^[^ ^76^ ^]^ However, individual atoms tend to aggregate into nanoclusters due to their high surface energy and thermodynamic instability.^[^ ^62^, ^63^, ^77^ ^]^ Loading SAs onto defect‐rich MXene through strong bonds effectively address agglomeration issues and maximize catalytic capacity. Strong interaction between Zn‐SAs doped MXene and sulfur makes possible Zn‐MXene@S cathode obtained just by aqueous solution mixing, which is much easier than the traditional melt‐diffusion method (Figure 4e).

Polymers, renowned for their abundance, diverse molecular structures, and well‐defined functional groups,^[^ ^54^, ^78^ ^]^ play a helpful role in flexible battery.^[^ ^54^, ^66^ ^]^ Moreover, the flexible nature of polymers allows them to accommodate high sulfur content and deform in response to the cathode's volume changes.^[^ ^61e^ ^]^ Wang et al. introduced a functional separator through vacuum filtration of a MXene and Nafion dispersion.^[^ ^64c^ ^]^ This uniform interlayer serves a cation permselective role, enhancing Li^+^ transport while arresting LiPSs through electrostatic repulsion. Thus, the organic/inorganic vdW heterojunctions formed by a combination of 2D MXene with semiconductor‐liked polymer films enable the fast charge‐transfer at the interface.

### 2.3 Inhibition of Shuttle Effect by Chemical Adsorption: Surface Groups

The external groups of MXene can be deliberately modified, and the group can significantly influence the observable properties of MXene. Professor Barsoum highlighted that the conductivity of MXene with hydroxyl or oxygens groups resembles that of transition metal carbides.^[^ ^80^ ^]^ To achieve specific external groups, various experimental techniques including chemical treatments, thermal annealing, and mechanical stripping processes have been employed.^[^ ^81^ ^]^ Additionally, MXene exhibits numerous reactive group sites, Ti atoms and polar groups on the surface, which results in strong chemisorption and a high affinity for Li–S intermediates. Hence, MXene demonstrates its immense potential in designing puissant chemisorption to alleviate the shuttling effect during C&D.^[^ ^37c^ ^]^

**Figure 5:**
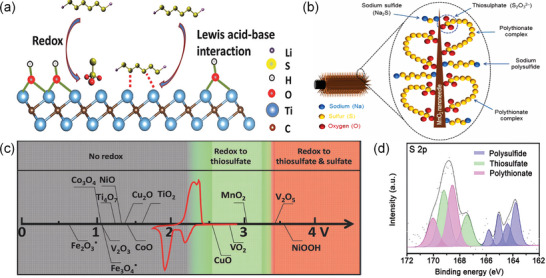
a) Schematic demonstrating the two‐step interaction between a representative hydroxyl‐decorated MXene phase and LIPSs. Reproduced with permission.^[^ ^89b^ ^]^ Copyright 2017, Wiley‐VCH. b) Illustration of the possible interactions of manganese oxide with different polysulfides and polythionate complexes. Reproduced with permission.^[^ ^91^ ^]^ Copyright 2019, Elsevier. c) Chemical reactivity of different metal oxides with LIPSs as a function of redox potential versus Li/Li. Reproduced with permission. Reproduced with permission.^[^ ^43^ ^]^ Copyright 2017, Wiley‐VCH. d) S 2p XPS profile of TiN@MXene after soaking Li_2_S_4_. Reproduced with permission.^[^ ^92^ ^]^ Copyright 2022, Springer Nature.

The shuttle effect, which concludes with continuous loss of active sulfur, discharge impediment and low utilization efficiency during the C&D process, poses a significant obstacle to the superb Li–S batteries. Soluble long‐chain LIPSs diffuses from the cathode to the anode, directly reacting with the lithium metal.^[^ ^82^ ^]^ To address this issue, researchers have focused on capturing and anchoring LIPSs, and have made prominent strides in physical confinement capture and chemical adsorption. Recent studies highlight the synergistic effect of their dual functional layers that MXene already possesses in composite‐based modifications.^[^ ^83^ ^]^ When it comes to physical confinement, the leading representative of non‐polar components is carbon materials, such as graphene,^[^ ^67^, ^83^ ^]^ CNTs,^[^ ^84^ ^]^ CNFs,^[^ ^85^ ^]^ and porous carbon fiber,^[^ ^86^ ^]^ act as a conductive network and a physical barrier. The adsorption force of physical capture is typically vdW force,^[^ ^87^ ^]^ which is so weak that the core issue of LIPSs shuttle movement remains unresolved. Chemical adsorption, achieved through polarized bonding by grafting functional groups on the MXene surface, serves as an predominant effective method for cathode interception and diaphragm anchoring of LIPSs.^[^ ^88^ ^]^ MXene provides a unique dual mechanism for effective chemical trapping of LIPSs, including novel conformational transition and Lewis acid‐base bonding interactions (**Figure** **5**a).^[^ ^89^ ^]^

#### 2.3.1 Configuration Transformation of Traditional Lithium Polysulfide

The classic configuration transition involves the conversion of Li_2_S_x_ (x = 3,4… 8) into less soluble thiosulfate and polysulfide complexes, thereby skipping the slow reaction of insoluble Li_2_S_x_ (x = 1,2) (Figure 5b). In 2015, the Nazar group proposed that the initial thiosulfate was produced by the oxidation of soluble LIPSs by active sites, such as external ‐OH groups on the host.^[^ ^90^ ^]^ Subsequently, the polysulfide complexes are accomplished by building up the newly formed long‐chain LiPSs, which are anchored by adsorptive S─S bonds of thiosulfates. With the reduction of long chains, short chains LIPSs can be obtained as the reactants for the next round of charging.^[^ ^91^ ^]^ Therefore, the absence of soluble LIPSs during discharge process effectively suppressed the shuttle effects (Figure 5c).^[^ ^43^ ^]^

XPS technology can be used to observe and analyze the sulfide configuration transition in the battery system. Wang et al. verified the coexistence of LIPSs, thiosulfate, and polysulfide through S 2p spectroscopy, demonstrating the strong interaction of titanium nitride with LIPSs (Figure 5d).^[^ ^92^ ^]^ Similarly, Yang et al. also found in the energy spectrum that the peak position of sulfur disappeared after cycling and instead formed thiosulfate and polysulfite complexes.^[^ ^67a^ ^]^ In addition, MXene can be used as a precursor for in situ synthesis of sheet layers to introduce external groups to other materials. For example, TiN@C based on this design was used for separator coating to suppress the LIPSs shuttle. The separator anchors LIPSs due to the interaction between thiosulfates, which also promotes sulfide conversion.^[^ ^83a^ ^]^ Moreover, the state‐of‐the‐art approach involves the combination of thiosulfate configuration transformation and catalysis of oxidation‐reduction reaction.^[^ ^93^ ^]^

#### 2.3.2 Lewis Acid‐Base Bonding Interactions

According to the general structural formula M_n+1_X_n_T_x_ of MXene,^[^ ^94^ ^]^ modifying the external functional group T has great potential for development. Therefore, the most straightforward optimization approach is to exchange the composition elements and types of external functional groups to increase the capture force on LiPSs. For example, Yuan et al. proposed a strategy of doping oxygen and sulfur into Ti_3_C_2_F_2_ to avoid insulating Li_2_S layer enveloping MXene.^[^ ^89c^ ^]^ In summary, the selection of corresponding elements is decisive for the adsorption effect.

Generally, TM and their compounds can be classified into a large group, such as unsaturated sites,^[^ ^93^, ^95^ ^]^ non‐Ti_3_C_2_ classes,^[^ ^89^, ^96^ ^]^ transition metal sulfides (TMS),^[^ ^45^, ^97^ ^]^ transition metal nitrides (TMN),^[^ ^92^, ^96^ ^]^ transition metal oxides (TMO),^[^ ^98^ ^]^ transition metal borides (TMB),^[^ ^96^, ^99^ ^]^ etc. Other classes are inorganic non‐metals (such as N‐doped C) and organic compounds such as pyrrolidine,^[^ ^88^ ^]^ dopamine,^[^ ^100^ ^]^ and conductive polymers.^[^ ^101^ ^]^

There is a weak binding of the TM nucleus to its outer electrons. Therefore, MXene is easy to forming polar dipoles. The sulfur in the LIPSs typically serves as an electron‐pair giver, that is, the Lewis bases, thus mostly forming coordination TM‐sulfur covalent bonds. The concept of unsaturated site design encompasses factors such as nonchemical coordination, low coordination and interfacial edges. For instance, low‐coordinated Ti bonds within 3D aerogels can yield active TiO_2_ catalytic sites.^[^ ^95a^ ^]^ Unsaturated coordination bonds can be artificially tuned on the layered edges of the MXene to obtain Ti‐S bonding with strong chemical interactions.^[^ ^95b^ ^]^ Furthermore, unsaturated Ti bonds with preserved end groups, such as O groups, showcase both chemisorption capabilities and catalytic activity.^[^ ^37c^ ^]^

Non‐Ti_3_C_2_ classification categories were using other elements to replace Ti and C sites. For instance, Mo_2_CT_x_ nanosheets enveloped by sulfur spheres exhibited MXene's chemisorption and high sulfur loading capacity.^[^ ^89a^ ^]^ Yu et al. illustrated that V_4_C_3_T_x_ facilitates the “adsorption‐diffusion‐transformation” process of LIPSs. Besides, there are molybdenum‐based Mo_2_B_2_‐MBenes.^[^ ^89b^ ^]^ By changing the elements, the unoccupied orbitals induced in the surface atoms act as Lewis acid sites with outstanding LiPSs capture ability.^[^ ^96a^ ^]^

**Figure 6:**
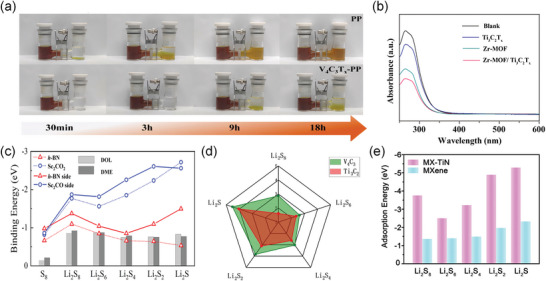
a) Schematic demonstrating the two‐step interaction between a representative hydroxyl‐decorated MXene phase and LIPSs. Reproduced with permission.^[^ ^96c^ ^]^ Copyright 2022, Elsevier. b) UV–vis absorption spectra of the Li_2_S_4_ solutions with the immersion of different materials. Reproduced with permission.^[^ ^74^ ^]^ Copyright 2021, American Chemical Society. c) The binding energies between Li_2_S_n_/S_8_ and different substrates. Reproduced with permission.^[^ ^104^ ^]^ Copyright 2021, Elsevier. d) Radar comparison chart of the adsorption energy of Ti_3_C_2_T_x_ and V_4_C_3_T_x_ for LIPSs. Reproduced with permission.^[^ ^96c^ ^]^ Copyright 2022, Elsevier. e) Adsorption energies of different LIPSs species on TiN@MXene and Ti_3_C_2_T_x_. Reproduced with permission.^[^ ^92^ ^]^ Copyright 2022, Springer Nature.

The investigation of shuttle effects employs widely recognized methodologies, including the H/I type permeation/adsorption experiment and DFT calculations. For the former, a dioxolane/ethylene glycol dimethyl ether (DOL/DME) co‐solvent electrolyte with reddish‐brown Li_2_S_n_ (n = 4 ≈ 8) solvent was loaded on the left side of an H‐type electrolytic cell, while pure electrolyte was placed on the right side. A functional separator was inserted in the middle to observe the ensuing color change (**Figure** **6**a). In the I‐type experiment, the modified electrode was immersed directly in the electrolyte for adsorption. Then the ultraviolet–visible spectroscopy (UV–vis) spectra were scrutinized to identify alterations in peak position and intensity, which reveals the concentration of S_4_^2‐^ (Figure 6b).^[^ ^102^ ^]^ The DFT approach, involving a combination of slab, layer, and other algorithms, was used to construct models, forecast the number of chemical bonds (Figure 6c) and elucidate adsorption energies (Figure 6d,e). Comparing these data helps to ascertain the anchored transition orientation of LIPSs. **Table** **1** compiles the characteristics of selected TM compounds, highlighting their distinctions. For instance, the bonding energy between LIPSs and VN (3.75 eV) significantly exceeds that with carbon‐based materials (1.07 eV), suggesting VN's superior affinity, and chemical uptake.^[^ ^103^ ^]^

**Table 1:** Performance comparison of transition metal based composite materials in Li–S batteries (X refers to non‐TM elements in TM compounds).

Materials	Application form	Electronegativity (TM‐X)	Features	Adsorption energy (Li_2_S_6_)	Capacity	Ref.
	MXene		Layered body, conductive network, active sites	1.407 eV		
TMS	CoS_2_‐Ti_3_C_2_O_2_	1.9–2.5	High binding energy, difficult synthesis process	3.05 eV	368.6 mAh g^−1^ (20C)	[84]
	TiS_2_	1.5–2.5	Lamellar structure, low conductivity	0.5 eV	739 mAh g^−1^ (100 cycles,0.2C)	[45b]
	VS_4_	1.6–2.5	Fine catalytic performance, poor chemical stability	0.91 eV	845mAh g^−1^ (100 cycles,0.14C)	[83b]
	SnS_2_	1.8–2.5	Volume expansion, high loading (6 mg cm^−2^)	0.69 eV	750mAh g^−1^ (100 cycles,0.2C)	[97b]
	MoS_2_‐ MXene	1.8–2.5	Limited adsorption ability	1.73 eV	408mAh g^−1^ (500 cycles,5C)	[97c]
TMN	TiN	1.5–3.0	High conductivity and cost	2.507 eV	1028mAh g^−1^ (100 cycles,0.2C)	[92]
	TiN‐C	1.5–3.0	High surface area	–	635.8mAh g^−1^ (600 cycles,1C)	[83a]
	VN	1.6–3.0	Polar bonds, easily oxidized	3.74 eV	1052.5mAh g^−1^ (250 cycles,0.1C)	[86]
	MoN‐CMK‐5	1.8–3.0	Excessive density	9 eV	658.4mAh g^−1^ (200 cycles,1C)	[96a]
TMO	TiO_2_	1.5–3.5	Ti‐S bonds, low conductivity	3.5 eV	513mAh g^−1^ (500 cycles,2C)	[98b]
	CeO_2_/MXene	1.1–3.5	High cost	2.234 eV	921mAh g^−1^ (200 cycles,0.5C)	[98a]
	SnO_2_	1.8–3.5	Small BET area	0.72 eV	301mAh g^−1^ (900 cycles,1C)	[105]
TMB	Mo_2_B_2_O_2_ (Mo_2_B_2_F_2_)	1.8–2.0	Low Li^+^ diffusion and decomposition barriers	2.2 (1.1) eV	–	[96b]
	Co_2_B‐CNT	1.9–2.0	Both Co and B can be bonded with S_4_^2−^	–	918mAh g^−1^ (3000 cycles,5C)	[106]
TMP	Ni_2_P	1.9–2.1	Li‐O‐P bonding, highly polarized Ni	1.55eV	626mAh g^−1^ (200 cycles,0.2C)	[40a]
	MOF‐CoP@MXene	1.9–2.1	Fine catalytic performance, volume change	–	797mAh g^−1^ (300 cycles,0.2C)	[107]

**Figure 7:**
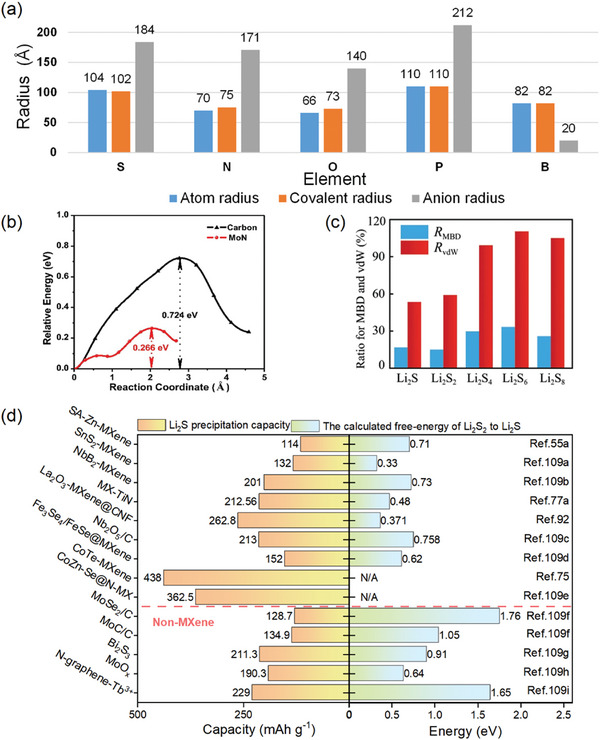
a) Histogram for the atom, covalent, and anion radius of anionic elements in commonly used TM compounds. b) Reaction energy barrier of Li_2_S decomposition harvested from carbon and MoN substrates. Reproduced with permission.^[^ ^96a^ ^]^ Copyright 2022, Elsevier. c) The ratio of MBD (R_MBD_) and vdW interaction (R_vdW_) on LIPSs in Ti_2_CF_2_ systems. Reproduced with permission.^[^ ^87^ ^]^ Copyright 2020, Springer Nature. d) Comparison of the catalytic effect of MXene‐based composites and other materials on polysulfides.^[^ ^55^, ^75^, ^77^, ^92^, ^109^ ^]^

TM compounds present a plethora of polar active sites for the absorption of LIPSs, which contributes significantly to substantial capacity retention in batteries.^[^ ^99^ ^]^ TMO compounds inherently display high polarization and function as ionic compounds with ionic bonding, effectively trapping dissolved LiPSs.^[^ ^97a^ ^]^ Sulfur has lower electronegativity than oxygen. Consequently, sulfur exerts reduced binding on Li^+^, and the larger radius of sulfur ions widens the transport channels for Li^+^ within the crystal (**Figure** **7**a). The negative S^2−^ or S_2_^2−^ ions enable positive metal atoms to possess high valence electron densities, creating binding sites for exogenous adsorbates like LiPSs. Additionally, the weak basicity of sulfur ions results in a covalent nature, enhancing the conductivity of TMS.^[^ ^99^ ^]^ Similarly, phosphorus can trap positively charged substances. Under appropriate atomic ratios of metal and P, TMP possesses metallic properties, and even superconductivity induced by strong electron delocalization can occur in the metal sublattice of phosphides.^[^ ^108^ ^]^ The nitrogen atom in TMN has electron lone pair electrons, so it can serve as a conductive Lewis substrate, promoting the formation of covalent bonds and enhancing the adsorption of Li_2_S_n_ (n = 4 ≈ 8).

To mitigate the shuttle effect, apart from the substantial adsorption onto LiPSs, there is also a synergistic cooperation with processes like Li^+^ transport, redox reactions, and site masking. Xiao et al. simulated that an excessively strong force of bare Mo_2_B_2_ on S_8_ could decompose it into S atoms and S_3_, disrupting multistage discharges.^[^ ^96b^ ^]^ Adding an external group to regulate the force within the proper range was suggested. The binding strength of Mo_2_B_2_O_2_‐MBene to LiPSs (2.4 ≈ 5.0 eV) surpasses Mo_2_B_2_F_2_‐MBene (1.0 ≈ 2.0 eV) without compromising catalytic efficiency, indicating potential for high‐rate Li–S batteries. Sometimes, doping, heterogeneous coatings can become a vicious blocking layer, thereby blocking the active sites of MXene. Introducing in‐plane mesopores or pores in the sandwich composite structure, such as those in the organic layer, enables more active substance storage and serves as a particle channel to expedite transport with low reaction barriers (Figure 7b).^[^ ^96a^ ^]^ Xu et al. employed a soft template interface self‐assembly strategy to prepare 2D‐mPmPD/MXene nanocomposites, where thin MXene nanosheets served as 2D coatings attached to the backbone.^[^ ^101b^ ^]^ This design mitigated the adverse effects of adsorption modification in ion mobility and Coulombic efficiencies.

Furthermore, there are insightful mechanisms to be explored in the anchoring process. The many‐body dispersion (MBD) method is a vdW force modification that takes into account the interactions between multiple dipoles. For Ti_2_CF_2_‐MXene systems with strong polarizability and vdW force‐dominated anchoring, the MBD effect significantly reduces the binding energy. Whereas for the N‐doped graphene system, which is chemically bonded dominated anchoring, the impact of the MBD effect is so delicate to be neglected (Figure 7c).^[^ ^87^ ^]^ Thus, when designing MXene‐based shuttle effect inhibitor components, it is inadvisable to blindly pile up the bonding values, and it is more sensible to judge the characteristics of each part and consider the feedback and collaborative cooperation between different structures.

Undoubtedly, the modification is not limited to grafting but can be combined with 3D structures, heterojunctions, catalysts, etc. Such as the binary synergistic MoS_2_/MXene cathode, wherein MoS_2_ nanosheets immobilizing LiPSs, heterointerface migrating tethered LiPSs to the MXene, then the plentiful interfacial oxygen terminals expediting the conversion kinetics.^[^ ^97c^ ^]^ In forthcoming investigations, excellent materials will be those with diverse composite functionality.

### 2.4 Catalytic Effect for Conversion of Polysulfide

Li–S batteries offer high energy density and low cost, but the shuttle effect and slow kinetics of LIPSs hinder their popularization as next‐gen energy storage systems. The sluggish chemical reaction occurs primarily due to the slow rate of the Li_2_S_2_ → Li_2_S conversion, as its Gibbs free energy (GFE) (ΔG = 0.6–1.4 eV) is around three to four times higher than that of the other steps, such as the Li_2_S_8_ to Li_2_S_4_ (ΔG = 0.01 ≈ 0.4 eV).^[^ ^110^ ^]^

Abundant active sites can be created to adsorb and promote the conversion of LIPSs by enhancing MXene materials through hetero‐structuring, doping and hybrid, grafting, in situ strategies, and other methods. As listed in Figure 7d, modified MXene materials generally exhibit reduced free energies from Li_2_S_2_ to Li_2_S and lower energy barriers from LIPSs to Li_2_S than non‐MXene materials. Furthermore, to investigate the catalytic abilities in the transition from LIPSs to Li_2_S, experiments were conducted to study the nucleation of Li_2_S through potentiostatic discharge at 2.05 V. All the modified MXene materials exhibit impressive capability for Li_2_S deposition, demonstrating enhanced catalytic effects on polysulfide species.

#### 2.4.1 Heterojunction Structure of MXene‐Based Materials

Currently, chemical heterojunction interface engineering is a commonly used catalytic strategy in Li–S batteries. This method creates unique interfaces that can improve the catalytic conversion kinetics of LIPSs. It has been observed that heterojunctions possess spontaneous built‐in electric fields (BIEF) which play an important role in facilitating the kinetic conversion of LIPSs. Additionally, these electric fields also aid in the transportation of electrons.

**Figure 8:**
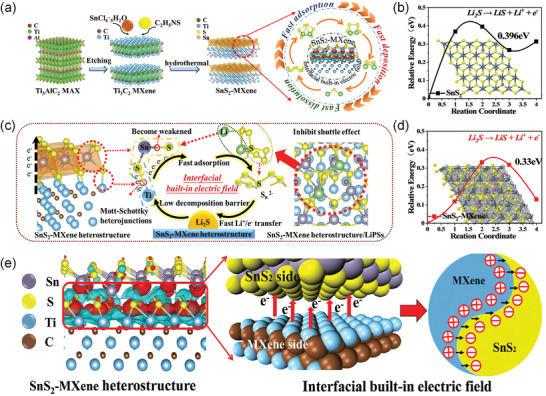
a) Schematic illustration of the synthesis processes and mechanism of SnS_2_‐MXene. b,d) The decomposition barrier and their corresponding top view of the Li_2_S decomposition path on the SnS_2_ surface and SnS_2_‐MXene heterojunction. c) Schematic illustration of the adsorption‐electrocatalysis mechanism of SnS_2_‐MXene heterojunction for the bidirectional sulfur redox. e) Schematic illustration of the electron flow direction simulation and interfacial BIEF. Reproduced with permission.^[^ ^109a^ ^]^ Copyright 2023, Wiley‐VCH.

Chen et al. synthesized a SnS_2_‐MXene Mott‐Schottky heterojunction catalyst with a BIEF at the interface by a one‐step hydrothermal reaction (**Figure** **8**a).^[^ ^109a^ ^]^ The presence of interfacial BIEF has a notable impact on the surface of SnS_2_ (Figure 8c), which results in the deposition of a surplus of electrons on the surface. These electrons tend to form chemical bonds with more LIPSs. During the nucleation or decomposition of Li_2_S, the ample supply of Li^+^ and electrons can rapidly move at the heterogeneous interface (Figure 8e). Subsequently, the simulation results for the decomposition barrier of Li_2_S on both the SnS_2_ and SnS_2_‐MXene heterojunctions are presented in Figure 8b,d. The lower decomposition barrier of Li_2_S on the SnS_2_‐MXene heterojunction (0.33 eV) compared to SnS_2_ (0.396 eV) is attributed to the formation of the BIEF.

**Figure 9:**
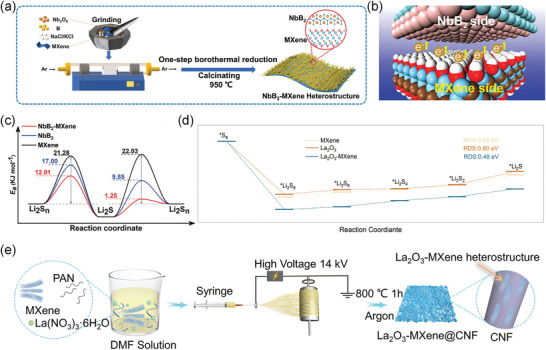
a) Synthetic scheme for NbB_2_‐MXene heterojunction. b) Schematics representing the electron redistribution at the interface between NbB_2_ and MXene. c) The activation energy for LIPSs/Li_2_S conversion on MXene, NbB_2_, and NbB_2_‐MXene, respectively. Reproduced with permission.^[^ ^109b^ ^]^ Copyright 2023, Wiley‐VCH. d) Schematic illustration for the La_2_O_3_‐MXene preparation process. e) Energy profiles for the reduction of LIPSs on La_2_O_3_, MXene, and La_2_O_3_‐MXene substrates. Reproduced with permission.^[^ ^77a^ ^]^ Copyright 2023, Wiley‐VCH.

Lu et al. prepared binary sulphidic NbB_2_‐MXene heterojunctions with BIEF (**Figure** **9**a). In which Nb and B atoms were bonded with LIPSs.^[^ ^109b^ ^]^ Spontaneous BIEF induces charge redistribution, directing more electrons towards NbB_2_ sites, thereby accelerating LIPSs transfer (Figure 9b). The calculation of activation energy (E_a_) was carried out to confirm that BIEF effectively lowers the diffusion barrier and reduces activation energy. It is noteworthy that NbB_2_‐MXene exhibits the lowest activation energy from Li_2_S_n_ to Li_2_S of 12.01 KJ mol^−1^ (Figure 9c). Besides, Huang et al. created a host for sulfur by developing a La_2_O_3_‐MXene@CNF heterojunction through electrospinning and carbonization (Figure 9e).^[^ ^77a^ ^]^ The La_2_O_3_ effectively capture dissolved LIPSs and MXene provide excellent conductivity. The as‐prepared La_2_O_3_‐MXene heterojunction demonstrates a significantly lower Gibbs free energy (0.48 eV) in comparison to La_2_O_3_ (0.80 eV) and the MXene electrode (0.68 eV) (Figure 9d).

In conclusion, heterojunctions of MXene‐based materials exert full synergistic effects by accelerating polysulfide transport and electron diffusion through a spontaneous BIEF. Under the joint action of heterojunctions and MXene materials, the conversion barrier of LIPSs is lowered, which stimulates complete discharge of the battery and enhances the cycle stability.

#### 2.4.2 Doping and Hybridization of MXene‐Based Materials

Elemental doping and substance hybridization are common methods for material improvement and can be applied in optimizing the catalytic transformation for LIPSs. Elemental doping generally alters the electronic structure and active sites of pristine materials, while substance hybridization synergizes the advantages of different materials to exhibit better performance. Elemental doping and hybridization of MXene‐based materials catalyze the nucleation/decomposition of Li_2_S and inhibit LiPS shuttling, making them well‐suited for Li–S batteries.

**Figure 10:**
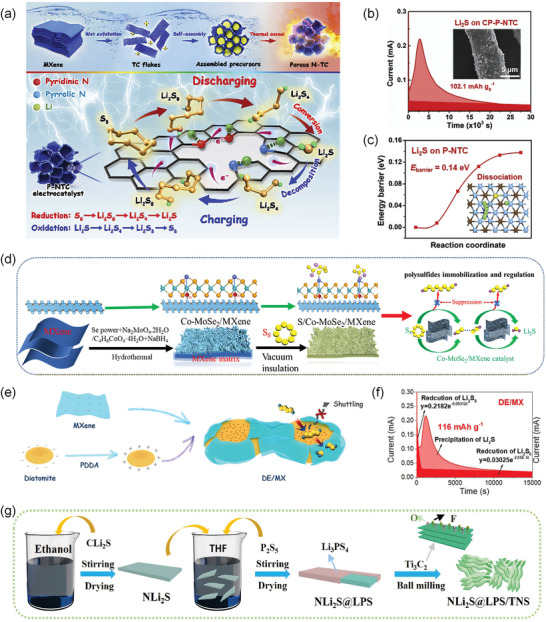
a) Schematic illustration of the MF‐templating synthesis of P‐NTC and its catalytic effect. b) Potentiostatic discharge profiles of Li_2_S_8_/tetralyme solution on CP‐P‐NTC substrates. c) Dissociation barriers and pathways of Li_2_S cluster on P‐NTC monolayers. Reproduced with permission.^[^ ^111^ ^]^ Copyright 2020, Elsevier. d) Schematic illustration of the Co‐MoSe_2_/MXene catalyst. Reproduced with permission.^[^ ^64a^ ^]^ Copyright 2021, American Chemical Society. e) Schematics of the synthesis of DE/MXene and regulation process of LIPSs on the DE/MXene interlayer. f) Potentiostatic discharge profiles of Li_2_S_8_/tetraglyme solution. Reproduced with permission.^[^ ^112^ ^]^ Copyright 2021, Elsevier. g) Schematic diagram of the fabrication process of NLi_2_S@LPS/TNS. Reproduced with permission.^[^ ^114^ ^]^ Copyright 2023, Elsevier.

In literature reports, TM and nitrogen are often exploited as dopants in MXene materials. Porous N‐doped Ti_3_C_2_ MXene (P‐NTC) was synthesized using the melamine formaldehyde (MF) template method (**Figure** **10**a).^[^ ^111^ ^]^ The synergistic effects of MXene and N doping enhance the conversion of LIPSs and decomposition of Li_2_S during C&D. According to Faraday's law, the calculated capacity of Li_2_S precipitated on CP‐P‐NTC reaches 102.1 mAh g^−1^ (Figure 10b). Furthermore, Song et al. assess the dissociation pathway and barrier of Li_2_S on P‐TNC substrates (0.14 eV) (Figure 10c).^[^ ^111^ ^]^ Wang et al. developed a bifunctional electrocatalyst, Co‐MoSe_2_/MXene nanosheets, by incorporating cobalt into MoSe_2_ (Figure 10d).^[^ ^64a^ ^]^ This modification enhances the catalyst's intrinsic catalytic activity and conductivity, which efficiently promotes sulfur conversion and prevents LIPSs shuttling.

Hybridization of other materials with MXene‐based materials as electrocatalysts allows synergistic effects to promote LIPSs conversion and kinetics. A hybrid electrocatalyst composed of 1D CoTe nanorods and 2D MXene materials was used as a separator modifier.^[^ ^75^ ^]^ The interplay within hybrid catalyzes the nucleation of Li_2_S and accelerates the conversion of Li_2_S_4_ to Li_2_S with low activation energy (1.8 kJ mol^−1^ lower than MXene). Fan et al. developed self‐assembled diatomite (DE)/MXene interlayers by using electrostatic interactions (Figure 10e).^[^ ^112^ ^]^ The potentiostatic discharge test presented the precipitation capacity of the Li_2_S precipitation on DE/MX is 116 mAh g^−1^ (Figure 10f). Some other composites, such as hollow core‐shell spheres of SiO_2_@Ti_3_C_2_T_x_,^[^ ^113^ ^]^ Li_2_S@Li_3_PS_4_@Ti_3_C_2_ (NLi_2_S@LPS/TNS) (Figure 10g), also exhibit rapid transport kinetics for Li^+^ and electrons, feature a low energy barrier, and demonstrate high catalytic activity.

#### 2.4.3 In Situ Strategies Based on MXene‐Based Materials

Currently, the formations of composites by combining with MXene materials through in situ strategies such as in situ growth, in situ sulfidation, in situ selenization, in situ crystal conversion, in situ nitridation, in situ formation, etc, are widely reported. These approaches enhance the transport of ions and electrons, expedite the conversion kinetics of LIPSs, and augment the number of active sites.

**Figure 11:**
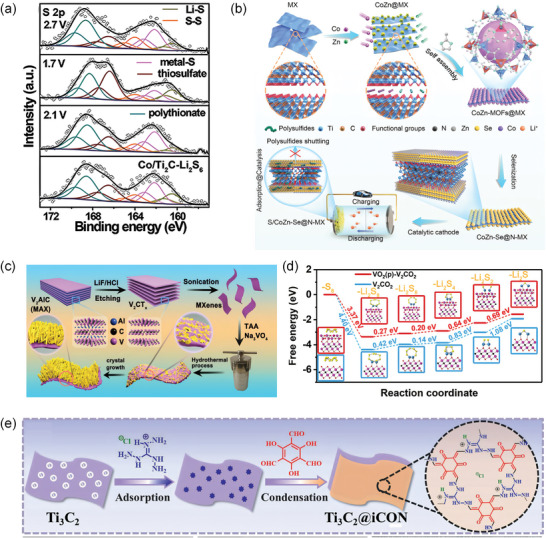
a) XPS spectra of S 2p on the Co/Ti_2_C‐Li_2_S_6_ at different discharge/charge states. Reproduced with permission.^[^ ^115^ ^]^ Copyright 2022, Wiley‐VCH. b) Synthesis process illustration of CoZn‐Se@N‐MXene. Reproduced with permission.^[^ ^109e^ ^]^ Copyright 2021, Wiley‐VCH. c) Diagrammatic representation of the preparation of VO_2_(p)‐V_2_C. d) Energy profiles for reducing LIPSs on VO_2_(p) and VO_2_(p)‐V_2_C. Reproduced with permission.^[^ ^116^ ^]^ Copyright 2019, American Chemical Society. e) Schematic illustration for the synthesis of Ti_3_C_2_@iCON. Reproduced with permission.^[^ ^73^ ^]^ Copyright 2021, Wiley‐VCH.

The in situ growth of cobalt nanoparticles on Ti_2_C nanosheets (Co/Ti_2_C) facilitates the enrichment of LIPSs and promotes electron transfer as well as Li^+^ replenishment at the interfaces.^[^ ^115^ ^]^ Remarkably, the reversible conversion of the LIPSs was verified by the high‐resolution XPS of S 2p. At the fully discharged state with a voltage of 1.7 V, the observable peak in the Li–S system is attributed to the conversion of LIPSs into Li_2_S. Conversely, at the fully charged state of 2.7 V, an inverse change phenomenon is observed (**Figure** **11**a). An innovative configuration involving the vertical growth of VO_2_(p) nanorod clusters on 2D V_2_C (MXene) nanosheets has been invented, serving as the sulfur hoster in Li–S batteries (Figure 11c).^[^ ^116^ ^]^ With simulations, the rate‐limiting conversion of Li_2_S_2_–Li_2_S has a low reaction barrier of 0.69 eV (1.08 eV, V_2_C) (Figure 11d). Li et al. utilized cooperative combining of Ti_3_C_2_ and iCON to establish novel avenues for mitigating the shuttle effect of LIPSs, accelerating the redox kinetics of sulfur species (Figure 11e).^[^ ^97a^ ^]^

In situ selenization methodologies are prevalent as well. To enhance the transformation of LIPSs, Fe_3_Se_4_/FeSe@MXene was synthesized for the first time by using self‐assembly and in situ selenization strategies.^[^ ^109c^ ^]^ Ye et al. synthesized 0D bimetallic selenide structures atop a 2D N‐doped MXene scaffold (denoted as CoZn‐Se@N‐MX) (Figure 11b) by an in situ colonization strategy.^[^ ^109e^ ^]^ The MXene nanosheets play a dual role as a catalytic substrate by promoting rapid electron and ion transport and preventing the aggregation of MOF‐derived selenides. Additionally, these selenides serve as effective spacers, isolating the MXene nanosheets and inhibiting restacking.

In situ strategies have also been applied to synthesize other compounds. For example, a one‐step in situ sulfidation process with thiourea was used to convert intrinsic Co nanoparticles on Ti_3_C_2_T_x_ to CoS_2_.^[^ ^27a^ ^]^ In addition, TiN@C sheets, V_3_S_4_@C and Ti_3_C_2_T_x_@CoNPs, were produced through the in situ nitridation, in situ crystal conversion and in situ growth strategy, respectively.^[^ ^83^, ^117^ ^]^ These approaches tend to avoid passivation of the active site, reduce the free energy of conversion of Li_2_S, and facilitate Li^+^/electron transport, thus accelerating the transformation of LIPSs and boosting capacity.

### 2.5 MXene‐Based Materials for Lithium Dendrite Inhibition

The emergence of lithium dendrites constitutes a pivotal factor with pronounced ramifications on the safety and longevity of Li–S batteries. Typically, the genesis of lithium dendrites can be ascribed to the nonuniform lithium flux and heterogeneous nucleation. Hence, the orchestration of charge distribution and the capacity to buffer volumetric alterations during the lithium plating and stripping processes emerge as pivotal determinants for the effective mitigation of lithium dendrite formation.

**Figure 12:**
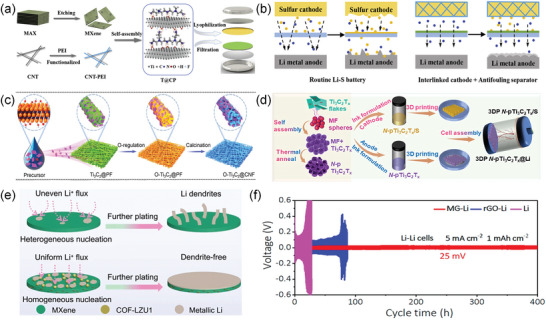
a) Illustration of the fabrication of T@CP nanohybrids for sulfur cathode and modified separator. b) Illustration for Li–S batteries with routine configuration and the proposed integrated configuration. Reproduced with permission.^[^ ^118^ ^]^ Copyright 2019, Elsevier. c) The mechanism schematic illustration of the O‐Ti_3_C_2_@CNF interlayer in the Li–S cell. Reproduced with permission.^[^ ^119^ ^]^ Copyright 2022, Elsevier. d) Schematic diagram illustrating the preparation process of the dual electrodes with the employment of N‐pTi_3_C_2_T_x_. Reproduced with permission.^[^ ^120^ ^]^ Copyright 2021, Elsevier. e) Schematics showing the mechanism of Li deposition on MF and MCF, respectively. Reproduced with permission.^[^ ^123^ ^]^ Copyright 2021, Springer Nature. f) High current density cycling of symmetric cells based on different electrodes with a stripping/plating capacity of 1 mAh cm^−2^. Reproduced with permission.^[^ ^48b^ ^]^ Copyright 2019, American Chemical Society.

Guo and colleague simplemented the one‐pot self‐assembly of Ti_3_C_2_T_x_ with polyethyleneimine (PEI) functionalized carbon nanotubes (T@CP) (**Figure** **12**a).^[^ ^118^ ^]^ In contrast to the conventional Li–S configuration, which is plagued by severe anode corrosion and unregulated growth of lithium dendrites resulting from flooded LIPSs and non‐uniform Li^+^ flux, the novel cell with its integrated design exhibited remarkable cathode stability and effective suppression of lithium dendrite formation (Figure 12b).^[^ ^118^ ^]^ Concurrently, the lipophilic O‐Ti_3_C_2_@CNF interlayer (Figure 12c) promote the even nucleation and growth of metallic lithium, and exhibit effects in promoting rapid interfacial electron transport and the preventing dendrite formation.^[^ ^119^ ^]^

**Figure 13:**
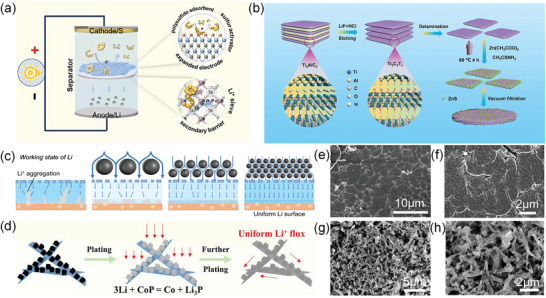
a) Schematic illustrating the Janus structure separator with different characteristics on two sides of cathode and anode in Li–S batteries. Reproduced with permission.^[^ ^121^ ^]^ Copyright 2023, Elsevier. b) Schematic showing the fabrication process of ZnS/MXene composite film. Reproduced with permission.^[^ ^122^ ^]^ Copyright 2023, Wiley‐VCH. c) The impact of V_2_C‐MXene size on the Li^+^ kinetics behavior. Reproduced with permission.^[^ ^51a^ ^]^ Copyright 2023, Wiley‐VCH. d) Interface mechanism of the heterojunction in Li deposition. Reproduced with permission.^[^ ^124^ ^]^ Copyright 2022, Elsevier. SEM images of the lithium electrode with MXene/ANF e,f) and Celgard 2400 separator g,h) after 450 h cycles with a stripping/plating capacity of 1 mAh cm^−2^. Reproduced with permission.^[^ ^127^ ^]^ Copyright 2023, Elsevier.

Robust framework can effectively inhibit lithium dendrite formation, addressing the specific requirements of advanced Li–S batteries. Nitrogen‐doped porous Ti_3_C_2_ MXene (N‐pTi_3_C_2_T_x_) framework can facilitate the deposition of Li^+^ while dispersing the local current density, effectively mitigating dendritic growth (Figure 12d).^[^ ^120^ ^]^ Meanwhile, MXene/COF framework (Figure 12e) and 3D MG framework effectively mitigate the formation of lithium dendrites by maintaining low overvoltage (Figure 12f). Liu et al. devised a slender Cu‐TCPP Janus separator, not only captures and prevents the movement of LIPS, but also serves as a discriminating Li^+^ sieve, contributing to the uniformity Li^+^ flux and lithium deposition (**Figure** **13**a).^[^ ^121^ ^]^ Wang et al. in situ grown uniform ultrafine ZnS nanodots on 2D Ti_3_C_2_T_x_ MXene nanosheets (Figure 13b).^[^ ^122^ ^]^ The presence of ZnS nanodots on MXene significantly enhances the adsorption capability of ZnS/MXene for both Li^+^ and LIPSs.

Composites of different materials often exert unexpected synergistic effects that are beneficial in promoting polysulfide conversion kinetics and inhibiting lithium dendrite growth. For example, Chen et al. boldly innovated in terms of structure, by introducing a concept of constrained structural design utilizing V_2_C‐MXene spheres, which serves the purpose of enhancing the structural ion sieving within Li–S batteries (Figure 13c).^[^ ^51a^ ^]^ CoP nanocages (CPNC) are intricately incorporated into the Ti_3_C_2_ nanosheets to form a Ti_3_C_2_/CPNC heterojunction, effectively mitigating the shuttle effect and lithium dendrites growth (Figure 13d).^[^ ^124^ ^]^ Pt‐SAs/In_2_S_3_/Ti_3_C_2_ heterojunctions strengthen the electronic effects at the heterogeneous interface, inhibiting the growth of lithium dendrites.^[^ ^125^ ^]^ A 2D MXene@COF heterojunction monoliths were used as lithium hosts, which exhibit extremely low overpotentials during lithium nucleation and deposition.^[^ ^126^ ^]^ Additionally, a MXene/aramid nanofiber (ANF) separator with “brick‐and‐mortar” microstructure was fabricated to creates a hierarchical ion path “highway.”^[^ ^127^ ^]^ The SEM images showcase a flat, soft, and dendrite free electrode surface (Figure 13e–h).

## 3 Potential Applications and Perspectives

**Figure 14:**
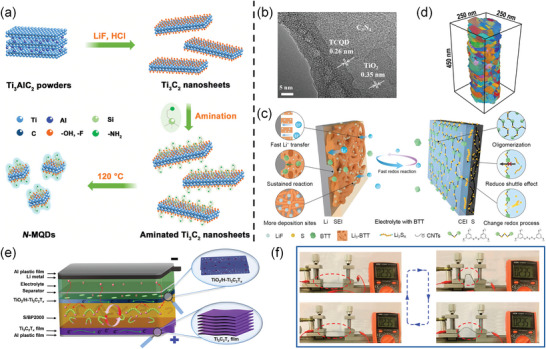
a) Synthetic route of the N‐MQDs. Reproduced with permission.^[^ ^136^ ^]^ Copyright 2022, American Chemical Society. b) HRTEM image of heterojunction. Reproduced with permission.^[^ ^137^ ^]^ Copyright 2020, Elsevier. c) Scheme for the Li–S battery with BTT electrolyte. Reproduced with permission.^[^ ^138^ ^]^ Copyright 2021, Springer Nature. d) 3D‐OMiTEM characterization of the deformed nano grained nickel. Reproduced with permission.^[^ ^139^ ^]^ Copyright 2023, American Association for the Advancement of Science. e) The flexible composite electrode has the Ti_3_C_2_T_x_ film as the current collector and TiO_2_/H–Ti_3_C_2_T_x_ as the coating layer. f) Demonstration of a flexible Li–S battery providing a stable OCV at different bend angles. Reproduced with permission.^[^ ^98b^ ^]^ Copyright 2022, American Chemical Society.

The 2023 Nobel Prize in Chemistry rewards the discovery and development of quantum dots (QD) and nanoparticles. QDs are very small, consisting of only a few thousand atoms. They are semiconductor nanostructures that bind excitons in three spatial directions. Their applications in energy conversion, catalysis, etc., stem from quantum effects.^[^ ^128^ ^]^ However, QDs are prone to clumping and can mask others, making it challenging to load active sulfur and resulting in poor cycling performance. The coupling between QDs with 2D materials can achieve a uniform distribution and eliminate agglomeration.^[^ ^129^ ^]^ The advantage of great surface proportion of MXene is further amplified by the QD.^[^ ^130^ ^]^ The efficient exchange or removal of functional groups within QDs decisively changes its structure and properties. In cutting‐edge superconductivity research, varying groups (e.g., Nb_2_C) lead to distinct superconducting critical temperatures and upper critical magnetic fields.^[^ ^131^ ^]^ The adsorption effect of QD introduces a novel concept of locally confining LIPSs, offering a solution to sheet aggregation and providing additional active sites for adsorption.^[^ ^132^ ^]^ QDs derived from MXene (MQD) inherit the excellent physicochemical properties of the parental MXenes and are easy to manufacture (**Figure** **14**a). In the high‐resolution transmission electron microscopy image presented in Figure 14b, the ultrathin three‐phase interface and the incorporation of MQDs contribute to an augmentation of charge transport channels. Accordingly, MQDs can be incorporated into the cathode as conductive and active additives, and the modification of the group opens up more possibilities, such as superconducting and photogenerated carriers.

Prefabricating and pretreating battery components represent effective methods for enhancing performance. Numerous researches have demonstrated the effectiveness of integrating preprocessed components before assembly, especially when they are meticulously designed and tailored under the guidance of scientific modification ideas.

Regarding the cathode:

- It can be filled with LIPSs. Wang et al. immersed carbon cloth (CC) and 1T‐MoS_2_ in the electrolyte.^[^ ^102^ ^]^ The solution became colorless and transparent after 12 h, that is, Li_2_S_6_ was completely adsorbed. At this point, the adsorbed electrodes were taken out and dried for 1 h and used as a cathode to assemble the cell.
- The doped heterogeneous materials can also be pretreated, such as prelithiated and amorphized. The utilization of lithiated 1T‐MoS_2_ (Li_x_MoS_2_) as a binder‐free conductive cathode enhances LIPSs adsorption. This introduces binding sites and establishes a conductive pathway for improved lithium translocation. Moreover, it serves as a lithium reservoir, contributing to higher kinetics.^[^ ^133^ ^]^ Amorphous materials present unique surface electronic states due to the long‐range disorder of the atoms. By modulating the amorphous arrangement, it is possible to achieve conformations that are conducive to the adsorption of LIPSs. Amorphization was employed to modulate the surface electronic states of cobalt oxide nanosheets.^[^ ^134^ ^]^ This resulted in the redistribution of d orbitals of cobalt atoms and an increase in unsaturated metal sites, which consequently enhances the binding energy with LIPSs. Hence, the prelithiation and amorphization of MXene would be groundbreaking.
- The material scheme for cathode can even be replaced. A battery with a Li_2_S/MXene cathode was assembled. Specifically, load Li_2_S on the V_2_CT_x_‐MXene as cathode, nudged elastic band calculations showcase its low migration barrier of Li^+^ (0.24 eV on V_2_CO_2_, 0.34 eV on V_2_C) and higher capacity retention.^[^ ^135^ ^]^ In general, MXene has been utilized as an adhesive‐free, self‐supporting structure for cathodes, meeting practical requirements for flexibility, lightweight design, and lithium storage.^[^ ^37a^ ^]^

Interfacial engineering plays a pivotal role in enhancing control over the sulfur reduction reaction (SRR). Making a simulated model for rate‐limiting SRR helps screening the better catalyst. The Li_2_S_4_ has recently been identified as a key intermediate in determining SRR kinetics.^[^ ^140^ ^]^ Regulating the terminal P‐band center of MXene and the electronegativity of its metasurface transition metal can influence the local chemical reactivity of MXene sites, thereby adjusting the SRR activity.^[^ ^141^ ^]^ Electrolyte additives provide the quickest method to modify the electrode's interface layer. For instance, the incorporation of 1,3,5‐benzene dithiol (BTT) as an additive facilitates the construction of SEI membranes on anode.^[^ ^138^ ^]^ The formed sulfur‐X (X = Li, S) bonds effectively regulate reversible Li deposition/detachment behaviors and modify redox pathways at cathode (Figure 14c). The utilization of BTT offering a straightforward technique rooted in bond chemistry. Heterogeneous interfaces are advantageous for electron‐accelerated reactions, and a direct correlation is observed between the p‐electron gain of sulfur in p‐block metal sulfides and the apparent activation energy (E_a_) of SRR, particularly on the LIPSs.^[^ ^142^ ^]^ This correlation underscores the fundamental principle behind heterojunction interface.

In some cases, advanced experimental methods contribute significantly to theoretical progress. In situ, liquid battery electrochemical transmission electron microscopy is instrumental in visualizing the atomic‐scale transformation of LIPSs at the electrode interface with high spatiotemporal resolution.^[^ ^143^ ^]^ This technique also investigates reactions and morphology evolution on various electrode surfaces. In Figure 14d, the use of 3D orientation mapping TEM technology allows for the observation of the nanoscale rotation of a single lattice through euler angle orientation mapping, providing an orientation map for a comprehensive understanding of changes during the reaction.^[^ ^139^ ^]^ Despite these advancements, battery research still exhibits unknownness, highlighting the ongoing need for innovative techniques to unveil the intricacies of electrochemical processes.

Antioxidant properties, safety, and stability present significant challenges in Li–S batteries, which have low tolerance to adverse factors such as heat and side reactions. In contrast to the current industry approach of using solid electrolytes for safety—characterized by high cost and reduced practical performance—MXene emerges as a cost‐effective, robust, and functional laminate material.^[^ ^144^ ^]^ MXene emerges as a cost‐effective, robust, and functional laminate material. The composite aerogel was fabricated through the co‐assembly of MXene and cellulose nanofibers using the freeze‐drying method. Subsequently, it underwent surface encapsulation with flame‐retardant thermoplastic polyurethane, which serves to enhance both the fire resistance and antioxidant capacity of the aerogel. Simultaneously, MXene forms robust hydrogen bonds with cellulose nanofibers, enhancing the mechanical elasticity.^[^ ^145^ ^]^ Safe pure Ti_3_C_2_‐MXene films were obtained via protonate colloidal treatment, devoid of foreign intercalators.^[^ ^31a^ ^]^ These films demonstrate stability in both water and oxygen atmospheres, with a doubling of conductivity compared to untreated counterparts. In addition, MXene can contribute to the improvement of the absorption shielding of electromagnetic waves by inducing dipole polarization through its surface functional groups and defects.^[^ ^145^ ^]^

Flexible wearable devices also require stabilization.^[^ ^146^ ^]^ In Figure 14e,f, a large area self‐supporting MXene is prepared using the drop‐coating method. The cathode consists of an Al foil‐Ti_3_C_2_T_x_‐S@C‐TiO_2_@Ti_3_C_2_T_x_ composite, which improves adhesion to the sulfur layer in flexible batteries.^[^ ^98b^ ^]^ The electrode is not dislocated or dislodged under bending, and the voltage remains unchanged. Self‐healing flexible batteries that are resilient to external interference are realized by coupling an encapsulated polymer with self‐healing function and phase change material.^[^ ^146a^ ^]^

Generally, the safety system battery design has the following ideas: (i) Structural safety: introducing a supporting framework during the synthesis of MXene and controlling the external groups; (ii) Working safety: coating a flame‐retardant and oxygen‐retardant modification layer on the flame; (iii) Safety management: self‐stabilizing repair materials that can autonomously cope with the outside impact and accidents.

## 4 Conclusion

In this review, we delve into the advantages of MXene as a functionalized Li–S battery component. Firstly, MXene demonstrates outstanding electrical conductivity, ensuring a swift movement of electrons and Li^+^. Secondly, its unique structure, such as a 2D large area to volume ratio and easy synthesis of heterojunctions, brings diverse transformative possibilities. The fusion of MXene with items like MOF, COF, TM composite will be a win–win strategy. Furthermore, we discuss the inherent mechanism of LIPSs anchoring and the design of external groups that effectively suppress the shuttle effect in Li–S batteries. MXene's surface conditions are easily modifiable, offering limitless potential. Its catalytic kinetic properties are summarized, namely expediting Li^+^ flux and promoting the deposition of Li_2_S. Lastly, we explore the prospective future applications of novel MXene in Li–S batteries by integrating the latest advances in various fields, including pretreating, superconductivity, superelasticity and safety. It is encourageous to discover open‐minded, sophisticated, and imaginative designs for more suitable MXene derivatives to guide composite strategy.

MXene has obvious functional advantages as a Li–S battery material. These properties lay the foundation for building high‐performance, safe, and reliable batteries. There is an urgent need to improve theory and understand the dynamic distribution, interfacial reactions, deposition and dissolution of LIPSs within Li–S batteries. The MXene‐based Li–S battery grounded in profound principles and scientific theory represents a significant step towards its commercialization.

## Conflict of Interest

The authors declare no conflict of interest.
